# Comprehensive Molecular Profiling for Precision Diagnostics: Integrating Genomic, Proteomic, and Epigenetic Landscapes in Human Pathology

**DOI:** 10.7759/cureus.116199

**Published:** 2026-09-13

**Authors:** Anandraj Vaithy K, Apoorv Shukla, Ajay Bhengra, Rakesh Venuturumilli, Falak Aara, Jigar Gusani

**Affiliations:** 1 Department of Pathology and Laboratory Medicine, All India Institute of Medical Sciences, Deoghar, Deoghar, IND; 2 Department of General Medicine, Jawaharlal Nehru Hospital and Research Centre, Bhilai, IND; 3 Department of Forensic Medicine and Toxicology, Shaheed Nirmal Mahto Medical College, Dhanbad, IND; 4 Department of Critical Care and Extracorporeal Membrane Oxygenation, Fortis Gleneagles Hospital, Hyderabad, IND; 5 Department of Microbiology, Sri Balaji Medical College and Hospital, Chennai, IND; 6 Department of Microbiology, Dr. N.D. Desai Faculty of Medical Science and Research, Dharmsinh Desai University, Nadiad, IND

**Keywords:** biomarkers, epigenomics, genomics, multiomics, proteomics

## Abstract

Comprehensive molecular characterization may enhance diagnostic assessment in complex human diseases by capturing biological heterogeneity beyond conventional single-layer approaches. This review examines the integration of genomic, proteomic, and epigenetic profiling for precision diagnostics and clinical interpretation. Major advances include comprehensive genomic characterization, high-throughput proteomic profiling, clinically informative epigenetic biomarkers, machine-learning-assisted data integration, and single-cell and spatial multiomics for resolving cellular and tissue-level heterogeneity. Evidence across diverse disease models indicates that integrated molecular data may enhance biomarker identification and provide additional information for disease classification, prognostic assessment, and prediction of therapeutic response; however, comparative measures of diagnostic performance are not consistently available. Proteomic and epigenetic information provides functional and regulatory context to genomic alterations, enabling a more complete representation of disease mechanisms. Current barriers include heterogeneous data formats, batch effects, missing data, limited standardization, high computational and financial requirements, privacy and regulatory concerns, and insufficient clinical validation. Future priorities should focus on harmonized analytical standards, reproducible pipelines, scalable computational infrastructure, improved model interpretability, robust clinical validation, and stronger interdisciplinary collaboration across oncology, rare genetic disorders, neurodegenerative disease, and other clinically heterogeneous conditions in routine practice. Integrative molecular profiling therefore represents a major direction for next-generation diagnostics, with its potential clinical value depending on analytical validation, clinical validity and utility, reproducible implementation, and prospective comparison with established diagnostic pathways.

## Introduction and background

High-throughput molecular technologies have transformed diagnostic pathology by complementing conventional morphology-based assessment with molecular classification systems that provide additional information on disease biology, prognosis, and therapeutic relevance. In oncology, the integration of tissue pathology with genomic profiling has provided additional molecular information for disease characterization, prognostic assessment, and therapeutic stratification [1]. Patient-specific molecular signatures increasingly inform diagnostic and therapeutic decisions [2].

Early developments were driven largely by genomic approaches, including mutation profiling, copy-number variation analysis, and targeted sequencing, which enabled the identification of disease-associated genetic alterations and clinically actionable mutations [3]. These advances have expanded genomic profiling from molecular characterization to treatment selection and patient stratification [3,4]. Genomic information alone, however, does not fully represent disease biology because important functional and regulatory processes occur downstream of DNA-level alterations. Protein expression, post-translational modifications, and epigenetic regulation can substantially influence cellular phenotype and disease behaviour [5]. Multiomics frameworks therefore integrate genomic, proteomic, transcriptomic, and epigenomic information to provide a systems-level representation of molecular processes [6,7]. Cross-validation of findings across molecular layers may also improve the robustness of candidate diagnostic and prognostic markers compared with reliance on isolated molecular measurements [8]. In colorectal cancer, such integrative approaches have demonstrated value for diagnostic, prognostic, and predictive molecular characterization [8].

The principal omics domains represent complementary levels of biological information. Genomics examines DNA-level alterations such as mutations, copy-number changes, and structural variants; transcriptomics evaluates patterns of gene expression; proteomics characterises protein abundance, modification, and interaction; and epigenomics examines regulatory processes including DNA methylation, histone modification, and non-coding RNA activity. Integrating these layers enables molecular alterations to be interpreted in relation to functional pathways and clinically observable phenotypes.

Clinical applications of integrated molecular profiling extend beyond solid tumours. In acute myeloid leukaemia, comprehensive omics analysis has improved characterization of the molecular landscape and facilitated translation of molecular findings into clinically relevant frameworks [9]. Similar approaches have supported risk assessment and therapeutic decision-making in renal cell carcinoma [10]. In gastrointestinal cancers, molecular imaging provides complementary phenotypic information that may support diagnosis, prognosis, treatment selection, and longitudinal monitoring alongside molecular profiling [11]. These examples illustrate how combining complementary molecular layers can generate clinically meaningful information that may not be obtained from individual omics platforms alone.

The increasing volume and dimensionality of molecular data have also created a major role for advanced computational approaches in diagnostic interpretation [12]. Machine learning (ML) and artificial intelligence (AI) can facilitate feature selection, predictive modelling, patient stratification, and assessment of therapeutic response across complex multiomics datasets [13]. Clinical implementation remains limited by differences in data formats and analytical platforms, insufficient standardisation, high costs, and the need for specialised computational infrastructure and expertise [14]. Regulatory requirements, data privacy, and the development of reproducible and validated analytical pipelines represent additional barriers to routine implementation [12]. Closer integration between omics research and clinical care is therefore necessary to translate molecular discoveries into actionable diagnostic information [6]. Next-generation diagnostic models are expected to combine genomic, proteomic, and epigenomic data with clinical information to support more accurate disease classification, prognostic assessment, and therapeutic decision-making.

Objective of the review

This review aims to examine how genomic, proteomic, and epigenetic profiling can be integrated to support precision diagnostics in human pathology. It also evaluates analytical validity, clinical validity, clinical utility, translational potential, and implementation challenges of multiomics approaches in disease classification, molecular marker identification, and therapeutic decision-making. Analytical validity refers to assay accuracy and reproducibility, clinical validity to the association with clinically relevant outcomes, and clinical utility to evidence of benefit in clinical decision-making or patient outcomes. Primary research studies are used, where available, to support claims concerning diagnostic performance, treatment response, prognostic utility, and clinical benefit, while narrative and review articles are used mainly to provide conceptual background, methodological context, and broader synthesis of developments in the field.

Methodology

This narrative review qualitatively synthesised evidence concerning the integration of genomic, proteomic, epigenomic, single-cell, spatial, and computational approaches in precision diagnostics. Relevant literature was identified through searches of PubMed, Scopus, Web of Science, and Google Scholar. The searches used combinations of terms related to “multi-omics,” “genomics,” “proteomics,” “epigenomics,” “precision diagnostics,” “molecular pathology,” “single-cell omics,” “spatial omics,” and “machine learning.” Boolean operators were used to combine the search terms, and the search syntax was adapted to each database's requirements. The literature search was intended to provide a broad and clinically relevant overview of the field rather than to constitute a formal systematic review or meta-analysis. Accordingly, study identification and selection were guided by relevance to the review objectives rather than by a Preferred Reporting Items for Systematic Reviews and Meta-Analyses (PRISMA)-based systematic screening framework. The database-specific search strategy is presented in Table 1.

**Table 1:** Database-Specific Search Strategy Used for Literature Identification

Database	Search strategy	Applied limits
PubMed	(“multi-omics” OR genomics OR proteomics OR epigenomics OR “single-cell omics” OR “spatial omics”) AND (“precision diagnostics” OR “molecular pathology” OR “precision medicine”) AND (“biomarker discovery” OR “disease classification” OR “therapeutic response” OR “machine learning”)	English-language, peer-reviewed publications relevant to human health
Scopus	TITLE-ABS-KEY (“multi-omics” OR genomics OR proteomics OR epigenomics OR “single-cell omics” OR “spatial omics”) AND TITLE-ABS-KEY (“precision diagnostics” OR “molecular pathology” OR “precision medicine”) AND TITLE-ABS-KEY (“biomarker discovery” OR “disease classification” OR “therapeutic response” OR “machine learning”)	English-language articles and reviews
Web of Science	TS=(“multi-omics” OR genomics OR proteomics OR epigenomics OR “single-cell omics” OR “spatial omics”) AND TS=(“precision diagnostics” OR “molecular pathology” OR “precision medicine”) AND TS=(“biomarker discovery” OR “disease classification” OR “therapeutic response” OR “machine learning”)	English-language articles and reviews
Google Scholar	“multi-omics” AND genomics AND proteomics AND epigenomics AND “precision diagnostics”; supplementary combinations included “single-cell omics,” “spatial omics,” “molecular pathology,” and “machine learning”	English-language scholarly publications screened for relevance

English-language, peer-reviewed publications related to human diseases, diagnostic applications, biomarker discovery, disease classification, prediction of therapeutic response, and clinically relevant approaches to multiomics integration were included. Only original research studies, translational investigations, methodological studies, and high-quality reviews were eligible for publication. Publications that were only available as conference abstracts, editorials, or commentaries, and those that duplicated other publications were excluded from the review, as were publications for which full text was not available. The literature selection process involved in the inclusion and exclusion process is shown in Table 2.

**Table 2:** Inclusion and Exclusion Criteria Applied During Literature Selection

Category	Inclusion criteria	Exclusion criteria
Language	Publications written in English	Publications written in languages other than English
Publication status	Peer-reviewed publications available in full text	Publications not available in full text
Population or disease context	Studies addressing human diseases or clinically relevant human pathology	Studies restricted exclusively to non-human models without clear clinical relevance
Subject area	Studies examining genomic, proteomic, epigenomic, multiomics, single-cell, spatial, or computational approaches	Studies unrelated to molecular profiling or precision diagnostics
Clinical relevance	Studies addressing diagnostic applications, biomarker discovery, disease classification, prediction of therapeutic response, or clinically relevant multiomics integration	Studies without diagnostic, translational, or clinically relevant applications
Publication type	Original research studies, Translational investigations, Methodological studies, High-quality review articles	Conference abstracts, Editorials, Commentaries, Duplicate publications
Evidence suitability	Publications containing sufficient information relevant to the objectives of the review	Publications containing insufficient information for qualitative interpretation

The titles and abstracts of identified publications were assessed for relevance to the objectives of the review, followed by full-text assessment where appropriate. Information was considered on the omics platforms, computational and analytical techniques, applicable disease areas, diagnostic and translational utility, and reported barriers to clinical implementation. Primary research findings were distinguished from secondary or review evidence during synthesis, particularly for clinically oriented claims, and primary studies were preferentially emphasized where available. Because this work was designed as a narrative review, formal study-selection counts, quantitative screening metrics, and a PRISMA flow diagram were not generated. The evidence was organised thematically across genomic, proteomic, epigenetic, computational, single-cell, spatial, and translational domains. Findings were synthesised qualitatively because of the methodological, technological, and clinical diversity of the literature. No meta-analysis, pooled effect estimation, or other statistical analysis was performed.

## Review

Theoretical framework of multiomics integration

The theoretical framework of multiomics integration is based on the hierarchical nature of biological information, which is represented by genomics, transcriptomics, proteomics, metabolomics, and epigenomics, which exist independently but are interrelated with the rest [15]. Genomics gives the blueprint of genetic information, which is then subjected to dynamic regulatory processes that finally determine the phenotype of a cell and the expression of a disease (downstream layers such as proteomics and epigenomics) [7]. The multi-level structure shows that there is a necessity to introduce various domains of omics to explain the entire range of biological complexity, in particular, in multifactorial diseases, where there are mechanisms of interaction between different molecular levels, which lead to the pathogenesis [5]. This is one reason why multiomics integration is based on a systems biology approach, which focuses on the holistic study of biological systems rather than individual components. This approach has been used to rebuild molecular networks, that is, recap the interaction between genes, proteins, and regulatory elements, and may be useful in better understanding the mechanisms and functional dysregulation in diseases [16]. They may be employed to assist researchers in discovering important regulatory hubs and signalling cascades that drive the development of disease, as well as enhance the interpretability of high-dimensional data [17]. Such integrative models have been particularly used in precision oncology, where intricate interactions of molecular processes determine tumour heterogeneity and variability in therapeutic responses [14].

Multiomics analyses involve high-dimensional and heterogeneous datasets that require advanced computational methods for integration and interpretation [15,18,19]. There are unsupervised learning models that can be used to identify the shared and modality-specific patterns in the datasets to derive biologically meaningful information, such as Multi-Omics Factor Analysis (MOFA) and others [18]. These approaches decrease the amount of data and retain valuable data, which enables the integration of different layers of omics to form a single model of analysis [15]. Normalisation of data, dimensionality reduction, and harmonisation of platforms are important steps towards ensuring the strength and reproducibility of the integrative analyses [19].

Some of the key challenges when incorporating multiple omics can include the fact that, in general, datasets are expected to be variable in size, resolution, and technical variability [15]. Another issue that complicates the analysis is noise, batch effects, and missing data, which should be overcome by the development of standardised pipelines and more powerful statistical models [19]. However, in spite of these challenges, integrative multiomics techniques have demonstrated colossal potential to improve disease classification, the discovery of new biomarkers, and targeted therapy by capturing the dynamic interaction of molecular layers [17]. More streamlining of computational tools and systems-level methodologies is likely to further streamline the theoretical framework of multiomics integration to be able to make even more precise and clinically relevant interpretations in human pathology [13]. An overview of the most important omics layers and their diagnostic potential is given in Table 3.

**Table 3:** Omics Layers and Clinical Relevance in Precision Diagnostics DNA: deoxyribonucleic acid; RNA: ribonucleic acid

Omics Layer	Key Features	Clinical Applications	Reference
Genomics	DNA sequence alterations, copy number variation, and structural variants	Cancer profiling, rare disease diagnosis, pharmacogenomics	[3]
Proteomics	Protein expression, post-translational modifications, and protein interactions	Biomarker discovery, therapeutic monitoring, disease stratification	[6]
Epigenomics	DNA methylation, histone modification, and non-coding RNA regulation	Disease regulation, diagnostic biomarkers, reversible therapeutic targets	[13]
Multiomics	Integrated analysis of genomic, proteomic, epigenomic, and clinical data	Systems-level disease interpretation and precision diagnostics	[15]

Genomic landscape in human pathology

Major Genomic Alterations

The genomic landscape in human pathology is a wide range of genetic alterations that collectively lead to the onset, progression, and response variability to treatment. The simplest and most basic form of genetic change is known as single-nucleotide variants (SNVs) and involves the replacement of a single base pair and can change the activity of proteins or regulate gene expression based on their location and biological context [3]. Other than SNVs, copy number variations (CNVs) play a significant role in controlling the dosage levels of a variety of genes through genomic amplifications or deletions and are thus involved in the oncogenic activation or silencing of tumour suppressor genes in most diseases [20]. Additional sources of genomic complexity are structural variants (such as deletions, insertions, inversions, and chromosomal rearrangements) disrupting the architecture of genes or forming new fusion genes with oncogenic potential [21]. All these various genomic changes have the overall effect of defining the molecular heterogeneity that can be observed in various pathological conditions, especially in cancers, where multiple co-existing changes define the molecular heterogeneity in that particular disease [5]. The advances in genomic technologies have enabled the characterisation of these developments holistically with improved resolution and accuracy as never before. With next-generation sequencing (NGS) systems, molecular diagnostics has been transformed by allowing high-throughput sequencing of complete genomes or areas of interest, making it possible to detect a wide range of genomic variants with a single assay [4].

Sequencing Technologies and Clinical Applications

Whole genome sequencing (WGS) is also a method that gives a global picture of the entire genomic landscape, which includes both coding and non-coding regions, whereas whole exome sequencing (WES) is a method that focuses on protein-coding regions, which harbour most clinically relevant mutations [3]. Instead, targeted gene panels offer a cost-effective and clinically viable solution because it focuses on a pre-defined collection of genes that are related to specific diseases and make quick and actionable decisions in routine diagnostics [12].

Liquid Biopsy and Molecular Residual Disease

More recently, liquid-biopsy approaches have expanded genomic profiling beyond conventional tissue-based analysis. Analysis of circulating cell-free DNA (cfDNA), including the tumour-derived fraction known as circulating tumour DNA (ctDNA), enables minimally invasive assessment of tumour-associated genomic alterations and facilitates longitudinal monitoring of molecular changes during treatment [8,14]. These approaches also have emerging relevance for detecting molecular residual disease (MRD), in which persistent or recurrent tumour-derived molecular signals may be identified after treatment before overt clinical progression becomes apparent [8,14]. Accordingly, cfDNA/ctDNA-based analysis represents an important contemporary extension of genomic diagnostics, with potential applications in treatment-response assessment, surveillance, and earlier identification of disease recurrence [8,14]. These technologies have been critical in defining disease-specific genomic profiles, with examples of clinical sequencing of metastatic colorectal cancer and sarcomas with diverse driver mutations and potential therapeutic targets [22-24]. Figure 1 illustrates a systems-level framework integrating genomic, transcriptomic, epigenomic, and proteomic information with computational analysis, clinical phenotype, validation, and clinical decision-making.

**Figure 1:**
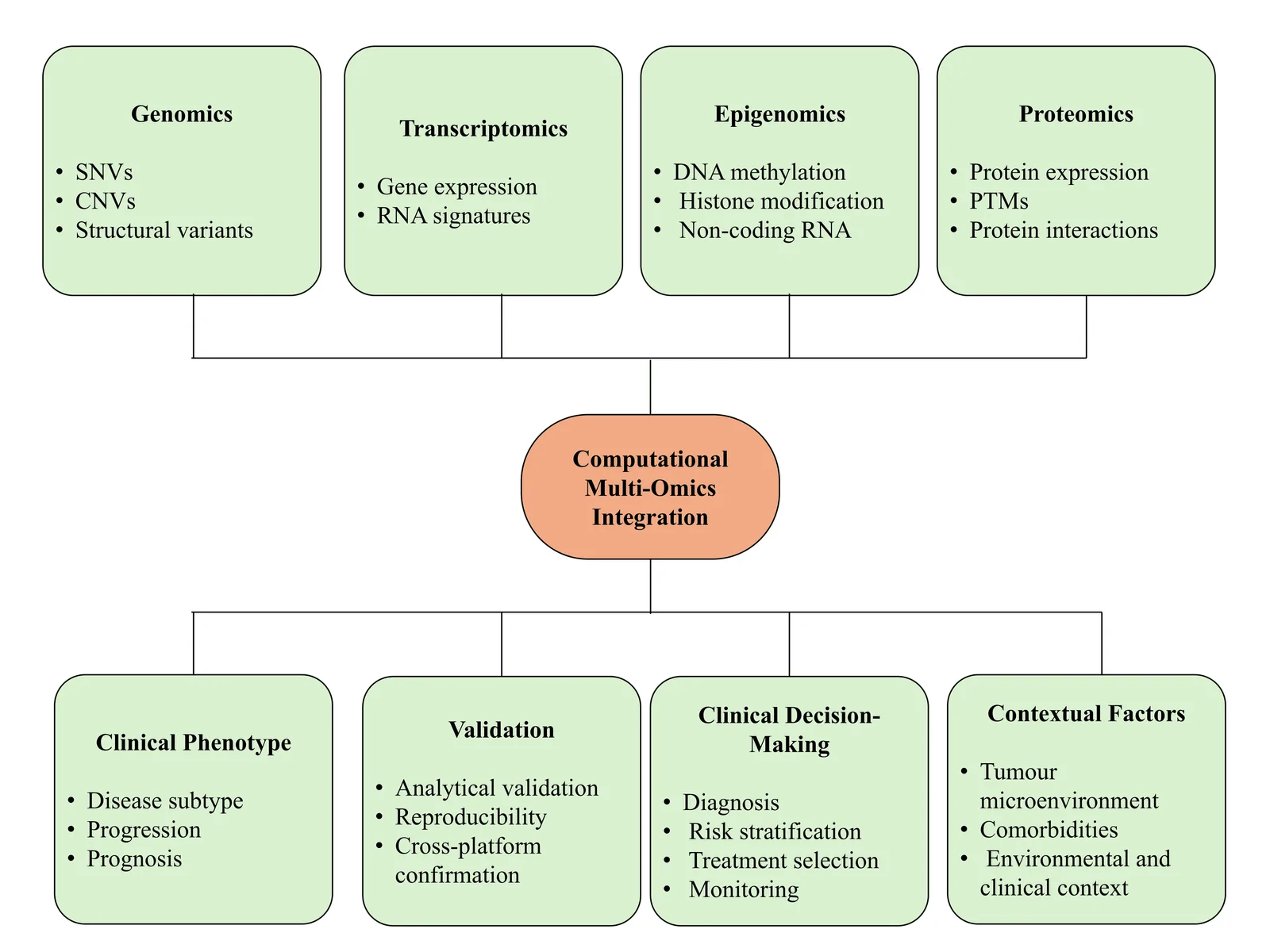
Systems-Level Integration of Multi-Omics Data for Precision Diagnostics and Clinical Decision-Making SNV: single-nucleotide variant; CNV: copy number variation; PTM: post-translational modification Created by the authors using Microsoft PowerPoint (Microsoft Corporation, Redmond, Washington, United States), based on sources [14-16].

Proteomic profiling and functional insights

Protein Expression and Functional Regulation

Proteomic profiling provides valuable functional data on the biology of diseases by determining dynamic protein expression, modification, and interaction that ultimately determine the cellular phenotype. The proteome is highly dynamic and situation-specific in contrast to the relatively fixed genome, which is in touch with real-time cellular states and influenced by environmental stimuli and regulatory mechanisms [25]. Post-translational modifications (PTMs), such as phosphorylation, acetylation, and ubiquitination, further augment the complexity of the proteome and regulate the activities of proteins, their stability, and their localisation, thus modulating key signalling pathways that play a role in disease pathogenesis [5]. In addition, protein-protein interactions form multifaceted molecular networks that control the functioning of cells, and their dysregulation is frequently associated with the development of disease and dysfunction of the cells [25]. It is now possible to systematically study protein interactions and stability to gain a better understanding of the molecular mechanisms underlying cellular activities and therapeutic targeting with innovative technologies such as thermal proteome profiling [26].

Proteomic Platforms and Clinical Translation

High-throughput, sensitive large-scale protein identification and quantification of thousands of proteins in complex biological samples rely on proteomics based on mass spectrometry [14]. Complementary affinity-based platforms have further expanded high-throughput proteomic profiling. Antibody-based platforms such as Olink (https://olink.com/) and aptamer-based platforms such as SomaScan (https://somalogic.com/somascan-platform/) enable multiplex measurement of large numbers of circulating proteins and have been increasingly applied in biomarker and population-scale proteomic studies [27]. Their clinical translation, however, requires rigorous analytical and clinical validation, including assessment of assay specificity, reproducibility, cross-platform concordance, independent cohort replication, and demonstration of clinically meaningful diagnostic or prognostic performance. Such validation is essential before proteomic signatures derived from high-throughput platforms can be incorporated reliably into routine diagnostic practice.

These technologies enable mapping the patterns of protein expression and functional states across the board and bridging the gap between the changes in the genome and the changes in the phenotype [6]. Additionally, new techniques, such as extracellular vesicle proteomics, allow minimally invasive profiling of circulating biomarkers, which opens up new possibilities to monitor and stratify diseases [28]. Proteomic profiling is increasingly gaining clinical significance in the discovery of biomarkers, classification of diseases, and monitoring of therapy. Disease-specific protein signatures have been discovered using proteomics and can be used as diagnostic and prognostic biomarkers across a spectrum of diseases, including neurodegenerative diseases such as Alzheimer's disease, where proteomic studies have revealed novel disease pathways that can be linked to disease progression [29].

Proteomic data can be utilised in cancer biology to further classify tumours and provide more personalised treatment options in addition to the earlier genomic data-based tumour stratification [8]. In addition to this, proteomic profiling can be used to measure therapeutic response by monitoring the changes in the expression of proteins and signalling pathways, and provide real-time updates on the treatment efficacy and mechanisms of resistance [13]. The fact that proteomics is part of multiomics systems further evidences the clinical relevance of proteomics by allowing more narratively and functionally relevant insights about the processes of a disease, ultimately supporting the development of precision diagnostics and personalised medicine [7]. Figure 2 outlines key proteomic mechanisms and clinical applications.

**Figure 2:**
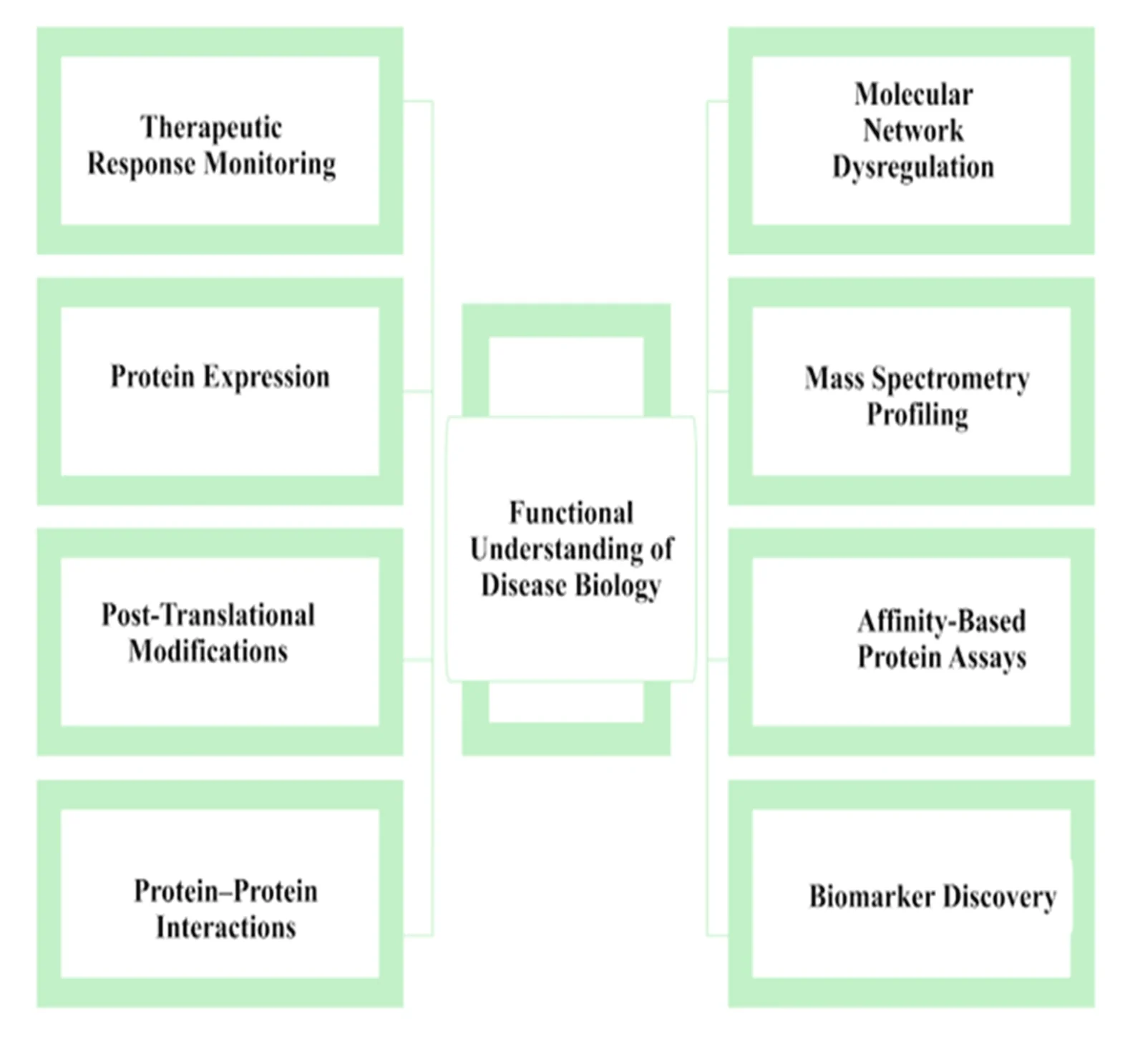
Proteomic changes revealing functional disease mechanisms and biomarkers Created by the authors using Microsoft PowerPoint (Microsoft Corporation, Redmond, Washington, United States), based on sources [25-27].

Epigenetic regulation in disease pathogenesis

The most significant layer of molecular regulation, which is involved in the pathogenesis of diseases and phenotypic variability, is epigenetic regulation. Epigenetic pathways that orchestrate the regulation of transcriptional activity and cellular identity include: DNA methylation, histone modifications, chromatin remodelling, and the control function of non-coding RNAs [30]. DNA methylation at CpG (cytosine-phosphate-guanine)-rich regulatory regions is an important epigenetic mechanism associated with transcriptional regulation and gene silencing [30,31]. The accessibility of chromatin and transcriptional dynamics is regulated by fine-tuned control of gene expression in response to environmental and cellular signals [31]. More complexity is added to epigenetic control through the further regulation of gene expression by non-coding RNAs, including microRNAs and long non-coding RNAs, further interacting with chromatin-modifying complexes, and further influencing post-transcriptional regulation [31].

Dysregulation in epigenetics is becoming increasingly identified as a major cause of a myriad of human disorders, such as metabolic disorders, neurodegenerative conditions, and cancer. The alteration in the epigenetics of the affected individuals in metabolic disorders influences the pathways involved in insulin signalling, lipid metabolism, and inflammation, thereby contributing to the development and progression of the disease [32]. Similarly, in neurodegenerative disorders, epigenetic processes are used to regulate neuronal gene expression and synaptic plasticity, with pathological aberrant modifications. Also contributing significantly to the pathogenesis of the disease are epigenetic changes caused by infection that form a dynamic relationship between the environmental factor and epigenetic regulation [33,34]. These observations indicate the role of epigenetic processes as a mediator between genetic predisposition and environmental factors in complex diseases [30].

The clinical potential of epigenetic profiling is great, especially when it comes to precision diagnostics and therapy. Epigenetic biomarkers, such as DNA methylation biomarkers and histone modification biomarkers, are increasingly finding their way to the diagnosis, prognosis, and even stratification of patients in a disease due to their stability and detectability in biological samples [14]. Unlike genetic mutations, epigenetic changes can potentially be reversed, and hence make attractive targets of therapeutic intervention using epigenetic drugs such as DNA methyltransferase inhibitors and histone deacetylase inhibitors [13]. The combination of the epigenetic information and the genomic and proteomic data will assist in better understanding the heterogeneity of a tumour and in developing a tailored treatment plan in the oncology field [12]. The fact that epigenetic landscapes are increasingly integrated into multiomics systems further emphasises their clinical utility, as they can give a more dynamic picture of the biology of disease, which can be better utilised in an increasingly wide range of clinical contexts [2].

Integration of multiomics data

The incorporation of multiomics data is based on complex computational methods that enable them to combine and analyse the heterogeneous biological information. An important preliminary step on the path to comparability across the various omics platforms, which are typically different in resolution, dimensionality, and technical noise, would be an important step of data preprocessing [35]. Systematic bias can be minimised through good preprocessing and may be used to enhance the reliability of later analyses, particularly in large-scale studies that involve genomic, proteomic, and metabolomic data [36]. The datasets can be combined using integrative approaches to analytical methods, either vertically by linking datasets to different layers of omics in the same sample, or horizontally by merging similar types of data across studies, thereby allowing wide biological interpretations [37]. Machine learning and artificial intelligence have become the frontiers in multiomics data integration, as well as providing scalability and efficacy frameworks to elicit meaningful patterns in the high-dimensional datasets. Algorithms of learning (supervised and unsupervised) help in feature selection, clustering, and predictive modelling to aid in disease classification and identification of biomarkers [38].

The strategies of deep learning, such as neural networks and autoencoders that capture complex nonlinear dependencies between multiple layers of omics data otherwise invisible to traditional statistical techniques, are also friendly to integration [39]. These methods have demonstrated promising discriminatory performance in selected oncology datasets; however, reported performance varies according to the disease, dataset, validation strategy, and molecular platform, and evidence from externally validated comparative studies remains necessary before superiority over established diagnostic approaches can be concluded [40]. Also crucial are the network-based models, which model the biological systems as a system of connected nodes and edges that facilitate the identification of key regulatory pathways and molecular interactions underlying the disease processes [38]. 

The clinical credibility of machine-learning models in multiomics analysis depends on rigorous model development and validation. Adequate sample size and events-per-feature should be considered relative to model complexity, with dimensionality reduction or feature selection used to reduce overfitting. Class imbalance should be appropriately managed, and strict separation of training and test data is necessary to prevent data leakage. Nested cross-validation can reduce bias during model selection and hyperparameter tuning, while performance assessment should include calibration in addition to discrimination. External validation in independent populations and independent replication are essential before high-dimensional models can be considered clinically generalisable. Recent reviews further emphasise that multiomics integration should be considered as a complete analytical pipeline encompassing preprocessing, dimensionality reduction, integration strategy, model development, performance evaluation, and interpretability [19]. AI-enabled multimodal approaches extend this framework by combining heterogeneous omics and phenotypic information, but their clinical credibility depends on rigorous validation and reproducibility [39].

The integration of multiomics is supported by the increasing ecosystem of bioinformatics platforms and computational infrastructures to work with and examine large-scale datasets. Integrative databases and software tools. It is possible to store, annotate, and cross-platform analyse data with the aid of integrative databases and software tools [36]. High-performance and cloud-based computing infrastructures provide scalable computational resources for processing and analysing large multiomics datasets [36]. The systems are required for the interpretation of multiomics information into clinically actionable information; in particular, high-quality and fast interpretation is critical in the context of precision diagnostics [12]. Although these developments have taken place, several issues still remain that continue to pose a challenge in the successful integration of multiomics. One of the biggest challenges that makes integration work difficult is the differences in types of data, format, and method of measurement [35]. Some biases can be caused by the batch effect and technical variation, and thus have to be extremely well corrected in their procedures [36]. In addition, the lack of standard guidelines and analysis techniques limits the degree of consistency and clinical applicability of multiomics techniques [5]. The key to the further integration of multiomics data and the discovery of the maximum potential of this technique lies in these problems [7]. Some of the key molecular technologies and calculations are shown in Table 4, that has been used to implement multiomics.

**Table 4:** Technologies and Computational Approaches for Multiomics Integration NGS: next-generation sequencing; WGS: whole-genome sequencing; WES: whole-exome sequencing

Category	Methods/Tools	Key Function	Reference
Genomic sequencing	NGS, WGS, WES, targeted gene panels	Detection of clinically relevant genomic alterations	[4]
Proteomic profiling	Mass spectrometry and interaction-based profiling	Functional protein mapping and pathway interpretation	[26]
Epigenomic analysis	DNA methylation and chromatin-regulatory profiling	Assessment of gene regulation and disease-associated epigenetic change	[31]
Machine learning	Supervised and unsupervised models	Pattern recognition, biomarker selection, and patient stratification	[38]
Deep learning	Neural networks and autoencoder-based models	Integration of high-dimensional multi-omics datasets	[40]

Single-cell and spatial multiomics

Single-cell and spatial multiomics technologies have become paradigm shifts in understanding the resolution of cellular heterogeneity and spatial organisation in complex biological systems, which has never been achieved before in precision diagnostics. Conventional bulk omics techniques mean average signals within heterogeneous cell populations and thus conceal important variations that can promote disease progression and therapeutic response [41]. Single-cell techniques overcome this limitation by enabling it to profile single cells and the identification of rare cell populations, lineage trajectories, and cell-specific molecular signatures that are key to understanding the complexity of disease [42]. This is particularly true when dealing with oncology, where intratumor heterogeneity is an important factor in the development and metastasis of tumours, as well as in resistance to therapy, which requires high-resolution analytical frameworks [14]. With the development of single-cell sequencing technologies, including single-cell RNA sequencing (scRNA-seq), single-cell ATAC-seq, and multi-modal platforms, it is now possible to interrogate multiple layers of molecules in individual cells simultaneously [41].

The technologies offer insights into gene expression, chromatin accessibility, and regulatory networks at the single-cell resolution, which would provide a more comprehensive understanding of the cellular states and transitions [43]. The microfluidics and droplet-based systems have been further refined to enhance throughput and scalability, enabling single-cell analysis to be even more accessible due to these advances [43]. Multiomics of single-cell data can be used to recreate the complex cellular ecosystem and how different cell types interact and form the basis of disease pathogenesis [42]. Spatial multiomics extends these opportunities by preserving the spatial context of the molecular data in the tissues and thereby linking the cellular functionality with the anatomical location. Spatial transcriptomics and proteomics technologies enable mapping of patterns of gene and protein expression in tissue sections directly, which allows understanding of the architecture and interactions of the microenvironment [41]. This aspect of space is vital in the study of diseases such as cancer, where the tumour microenvironment, including immune cell infiltration and stromal interaction, is critical in disease progression and response to therapy [12]. A combination of spatial and single-cell data allows the researcher to get a whole picture of the molecular heterogeneity at both cellular and spatial scales [42]. The uses of single-cell and spatial multiomics in precision diagnostics are quickly diversifying into various disease-related areas. The methods can be used in tumour biology to characterise tumour ecosystems, identify populations of resistant cells, and discover new therapeutic targets [13]. They also allow studying the tissue structure and interactions between cells in non-cancer diseases, which leads to a better understanding of pathological processes and the discovery of biomarkers [2]. The key component of next-generation diagnostics will become single-cell and spatial multiomics, as they will provide high-resolution and context-aware information about human pathology [41].

Translational applications in precision diagnostics

Translational applications of multiomics profiling are the core of the development of precision diagnostics, which can transform the information generated through the application of multiomics profiling into clinically actionable strategies in a variety of disease domains. In oncology, integrative tumour profiling combining genomic, proteomic, and epigenetic data can provide increasingly detailed molecular characterization and help identify actionable alterations, molecular subtypes, and therapeutic vulnerabilities [14]. However, greater molecular resolution does not itself establish clinical utility; prospective validation, cross-platform reproducibility, and evidence that molecular findings improve clinically relevant decision-making remain necessary before widespread implementation [14].

Complementary molecular imaging approaches may extend tissue-based multiomics by providing spatial and longitudinal phenotypic information. In gastrointestinal cancers, integration of molecular imaging with molecular profiling may support diagnosis, prognosis, treatment selection, and treatment-response monitoring [11]. This complementary approach illustrates how molecular and imaging phenotypes may jointly contribute to precision diagnostics, although their incremental clinical benefit requires further comparative validation.

Multiomics allows for a better understanding of the heterogeneity and evolution of tumours, helping to better stratify patients and choose treatment based on the unique characteristics of the tumour [13]. Disease-specific examples further demonstrate this translational value. In breast cancer, human epidermal growth factor receptor 2 (HER2) amplification guides HER2-directed therapy, while *PIK3CA* mutations may support PI3K-targeted treatment. In non-small-cell lung cancer, *EGFR* mutations, *ALK* rearrangements, and *ROS1* fusions inform genotype-directed therapy. In colorectal cancer, microsatellite instability/mismatch-repair deficiency supports immunotherapy selection, whereas* KRAS* status influences anti-epidermal growth factor receptor (EGFR) treatment. In gliomas, IDH mutation status contributes to molecular classification and prognosis, while MGMT promoter methylation provides information relevant to alkylating chemotherapy response. These examples show how molecular alterations can directly inform diagnosis, prognosis, and therapeutic decision-making.

The introduction of the technologies of liquid biopsy, including circulating tumour DNA (ctDNA) and extracellular vesicle analysis, has also contributed to the broadening of the diagnostic potential due to the possibility of minimally invasive monitoring of tumour dynamics and response to treatment [8]. These techniques can provide longitudinal information on disease progression and treatment response and may support clinical decision-making; demonstration of improved patient outcomes, however, requires prospective comparative evidence establishing clinical utility [3]. Besides the oncology community, multiomics integration is on the rise in the neurological disease field, and complex molecular events underlie the pathogenesis and clinical heterogeneity.

Single-cell and spatial multiomics technology is a high-resolution profiling technique that can be used to identify cell-specific molecular signatures that can be associated with neurodegeneration, providing new insights into the pathogenesis of diseases [42]. Proteomic and transcriptomic methods have been instrumental in uncovering biomarkers that can be employed to help in early diagnosis and monitoring of disease, which is a dire unmet need under such circumstances as Alzheimer’s disease and other neurodegenerative diseases [44]. These advances are applied to come up with a specific therapeutic intervention by offering a relationship between molecular alteration and functional outcome in the neural tissues [45].

Multiomics can be used as a comprehensive approach to study the interaction of the host-pathogen and immune response in infectious and inflammatory diseases. The profiling of both host and microbial factors enables the identification of molecular pathways that mediate infection, immune activation, and disease progression [6]. This systems-level of knowledge is essential in the development of particular therapies and improved diagnostic accuracy in complex inflammatory diseases [12]. Immune profiling can be carried out using multiomics data and can be used to characterise the population of immune cells, signalling networks, and cytokine responses, which can be used to identify biomarkers that predict the severity of disease and the response to treatment [13]. The construction of computational biology and machine learning that enable the combination and analysis of complex data has increasingly supported clinical applications of multiomics data [46]. These technologies enable predictive modelling and decision support systems that can increase the accuracy of diagnosis and optimal therapy [47].

Multiomics-driven translational research is still the way to overcome the divide between molecular discovery and patient care. Hopefully, this transfer of such techniques into clinical workflow will further facilitate precision diagnostics by being able to offer more accurate, personalised, and dynamic disease management plans in a variety of medical fields [45].

Table 5 outlines the clinical applications of multiomics profiling within important disease areas.

**Table 5:** Translational Applications of Multiomics in Human Diseases

Disease Area	Multi-Omics Contribution	Clinical Impact	Reference
Oncology	Integrated tumour profiling and molecular subtype identification	Targeted therapy selection and precision oncology	[14]
Colorectal cancer	Diagnostic, prognostic, and predictive biomarker integration	Improved patient stratification and treatment planning	[8]
Acute myeloid leukemia	Comprehensive omics-based molecular landscape analysis	Better disease classification and clinical translation	[9]
Renal cell carcinoma	Integrated molecular profiling of tumour biology	Risk assessment and personalised therapeutic direction	[10]
Neurodegenerative disease	Proteomic and epigenetic biomarker discovery	Earlier diagnosis and disease monitoring	[29]

Limitations and future directions

This narrative review has several limitations. The search was restricted to English-language publications, potentially introducing language and publication bias. Heterogeneity in diseases, omics platforms, analytical methods, and study designs precluded quantitative synthesis, and inclusion of both primary studies and reviews may have affected evidence independence. No formal risk-of-bias or quality-appraisal framework was applied. Database overlap may have led to repeated identification of the same studies, while the broad narrative scope may underrepresent certain diseases, populations, and clinical settings. Rapidly evolving computational platforms and analytical pipelines may also limit the long-term applicability of some conclusions. Quantitative measures such as sensitivity, specificity, AUC, calibration, and decision-curve benefit were inconsistently reported; therefore, analytical or clinical validity should not be interpreted automatically as clinical utility.

Integrated genomic, proteomic, and epigenetic profiling provides a valuable systems-level framework for molecular interpretation, disease classification, prognostic assessment, and therapeutic decision-making. Future research should prioritise prospective validation, harmonised analytical standards, cross-platform reproducibility, transparent computational pipelines, and clinical utility assessment. Established patient-level benefit remains uncertain because comparative effectiveness and cost-effectiveness evidence is still limited.

## Conclusions

Integrated genomic, proteomic, and epigenetic profiling provides a systems-level framework that may support more comprehensive disease classification, prognostic assessment, and therapeutic decision-making in precision diagnostics. Compared with isolated single-omics approaches, multiomics integration enables more comprehensive interpretation of molecular alterations and their functional and clinical relevance. Computational approaches, including machine learning and network-based models, increasingly support the analysis of complex, high-dimensional datasets, while single-cell and spatial multiomics technologies provide higher-resolution characterization of cellular and tissue-level variation. Despite these advances, routine clinical implementation remains constrained by analytical heterogeneity, limited standardisation, computational requirements, cost, regulatory considerations, and the need for robust clinical validation.

Future progress should therefore prioritize reproducible analytical pipelines, cross-platform harmonisation, prospective validation, and effective integration of molecular findings with clinical workflows. Clinical adoption should depend not only on analytical validity and reproducibility but also on demonstrated clinical validity and evidence of clinical utility relative to established diagnostic pathways. The specific contribution of this review is its integrated consideration of genomic, proteomic, epigenetic, computational, single-cell, spatial, and translational dimensions within a single precision-diagnostics framework, with particular emphasis on distinguishing analytical validity, clinical validity, and clinical utility. This broader synthesis differs from reviews focused primarily on individual computational pipelines, specific omics platforms, or selected disease settings. With these requirements addressed, multiomics approaches may support more precise and clinically actionable diagnostic and therapeutic strategies, although demonstrable patient benefit and cost-effectiveness require further prospective validation.
